# Mechanical Behavior of Undoped n-Type GaAs under the Indentation of Berkovich and Flat-Tip Indenters

**DOI:** 10.3390/ma12071192

**Published:** 2019-04-11

**Authors:** Lixia Xu, Lingqi Kong, Hongwei Zhao, Shunbo Wang, Sihan Liu, Long Qian

**Affiliations:** 1 School of Mechanical and Aerospace Engineering, Jilin University, 5988 RenMin Street, Changchun 130025, China; xulixia@jlu.edu.cn (L.X.); 15044146899@163.com (L.K.); wang.shun.bo@163.com (S.W.); liusihan0528@163.com (S.L.); qianlong17@mails.jlu.edu.cn (L.Q.); 2 Key Laboratory of CNC Equipment Reliability, Ministry of Education, Jilin University, 5988 RenMin Street, Changchun 130025, China

**Keywords:** mechanical behavior, undoped n-type GaAs, Berkovich indenter, flat-tip indenter, machining tool

## Abstract

In this research, the mechanical behavior of undoped n-type GaAs was investigated by nanoindentation experiments using two types of indenters—Berkovich and flat-tip—with force applied up to 1000 mN. From the measured force-depth curves, an obvious pop-in phenomenon occurred at force of 150 mN with the flat-tip indenter representing elastic–plastic transition. The Young’s modulus and hardness of GaAs were calculated to be 60–115 GPa and 6–10 GPa, respectively, under Berkovich indenter. Based on the observation of indent imprints, the fracture characteristics of GaAs were also discussed. A recovery of crack by the next indentation was observed at 1000 mN with Berkovich indenter. In the case of flat-tip indentation, however, surface material sank into a wing shape from 400 mN. In this sinking region, a density of fork-shaped sinking, slip lines, and crossed pits contributed to the slip bands, and converging crossed twinning deformations inside the GaAs material were generated. Since cracks and destructions on GaAs surface took place more easily under the flat-tip indentation than that of Berkovich, a machining tool with a sharp tip is recommended for the mechanical machining of brittle materials like GaAs.

## 1. Introduction

As one of the most important III–V compound semiconductors, gallium arsenide (GaAs) has many applications in optical and electronic devices [1,2,3]. To satisfy the increasingly high-quality product requirement, lots of research has been devoted to the study of crystalline structural, electronic, optical, as well as mechanical properties [4,5,6,7,8] of GaAs. Nanoindentation is a simple yet powerful technique allowing to measure elastic modulus, hardness, and plasticity of materials at nanoscale, thus wildly applied to investigate mechanical properties of semiconductors [9,10,11,12,13]. Li et al. [9] researched the plastic deformation and phase transformation of GaAs single crystal under indentation with the loads of 0.0049, 0.049, and 0.098 N. Chang et al. [10] investigated the nanomechanical characteristics of Si and GaAs by nanoindentation and nanoscratch techniques. In the study, the hardness, Young’s modulus, and plastic energy were calculated from the indentation loading-unloading curve. Pirouz et al. [11] studied the temperature dependence of mechanical properties of undoped GaAs using the microindentation technique. The brittle-to-ductile transition temperature was estimated and dislocation configuration in the plastic zone was also discussed. Wasmer et al. [12] observed dislocations, twins, and slip bands in GaAs under nanoindentations and nanoscratches with Berkovich and wedge indenters via transmission electron microscopy. The difference in microstructural deformation is caused by the crystallography and indenter tip geometry. Klinger et al. [13] measured the hardness and Young’s modulus for several III–V compound semiconductors in a wide compositional range using a Berkovich indenter.

Due to the diverse indentation conditions—such as the load level, loading and unloading rates, tip geometry of used indenter, temperature, etc.—the obtained results from different literatures vary largely. Furthermore, in most studies, the maximum applied force was limited to be about 100 mN focusing on the investigation of nano-/micro-mechanical characteristics of GaAs. The objective of this paper is to research the mechanical behavior of GaAs during nanoindentations of Berkovich and flat-tip indenters with force from 50 mN up to 1000 mN. From the indentation loading-unloading curves and scanning electron microscopy (SEM) images of indent imprints, Young’s modulus, and hardness were calculated and the elastic-plastic deformation as well as fracture characteristics were discussed, respectively.

## 2. Experiments

**Figure 1:**
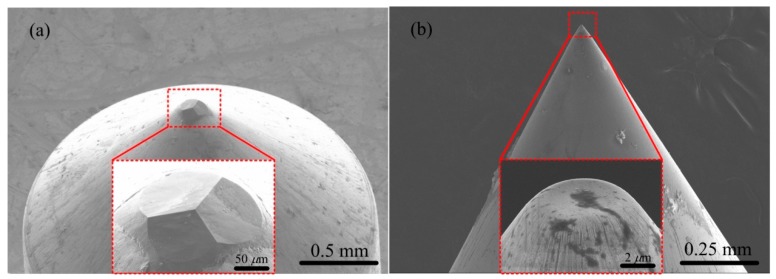
Scanning electron microscopy (SEM) images of (**a**) Berkovich, and (**b**) flat-tip indenters by HITACHI S-4800 (Tokyo, Japan). The insets in (**a**,**b**) show the enlarged images of the indenter tips, respectively. The pyramid lengths of Berkovich indenter in (**a**), with a facet angle of 65.3 degrees, are 62, 66, 75 μm, respectively. The radius of flat-tip indenter in (**b**) is 2.5 μm with a cone angle of 60 degrees.

**Figure 2:**
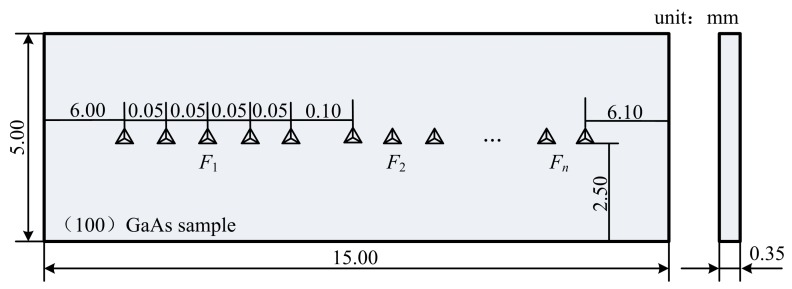
Schematic of the indent distribution on the GaAs sample (100) surface by Berkovich indenter (the size in the figure is not in scale). *F*_i_ represents the indentation force. The size of the sample is 15.00 mm in length, 5.00 mm in width, and 0.35 mm in thickness. The indents are distributed along the mid-width line with the distances of about 2.50 mm and 6.00 mm away from the four edges of the sample. The distance between the indents under the same peak force is 0.05 mm, and the distance between the neighboring indents under different forces is 0.10 mm.

Nanoindentation experiments were performed using our indentation system [14] at room temperature with two types of diamond indenters: a Berkovich indenter and a flat-tip indenter. The SEM images of these two indenters are shown in Figure 1. The tested sample material in the experiments was undoped n-type GaAs (100) provided by MTI Corporation in Hefei, China. Two pieces of samples were prepared with each sample indented by Berkovich and flat-tip indenters, respectively. The size of each sample was 15.00 mm in length, 5.00 mm in width, and 0.35 mm in thickness, as illustrated in Figure 2. The indented surface of the sample was polished with to a roughness less than 15 nm.

Before experiments, fused quartz from Hystitron Inc. was indented to calibrate the testing device according to the reference-mapping method [15]. After the calibration check of the device, Berkovich indentations were carried out with peak forces from 50 to 1000 mN, while the flat-tip indentations from 50 to 800 mN. In each single indentation experiment, the operating times were set to be 40 s for loading, 5 s for retaining at peak force, and 40 s for unloading. For each peak force level, five repeated indentations were made with 50 μm distance between adjacent indents. Under different peak forces, the distance between neighboring indents was 100 μm, as shown in Figure 2. All the indents were distributed on the indented sample surface along the mid-width line with the distances of about 2.50 mm and 6.00 mm away from the sample edges as illustrated in Figure 2. These spacing distances were large enough for each set of data to be unaffected by deformation resulting from nearby indents with peak forces of ≤800 mN for Berkovich indenter and ≤500 mN for flat-tip indenter (see Figure 5), respectively. After experiments, the indented samples were observed via SEM (HITACH S-4800, 5.0 kV).

## 3. Results and Discussion

**Figure 3:**
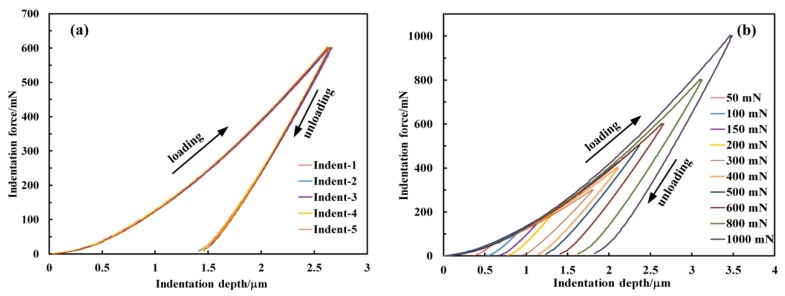

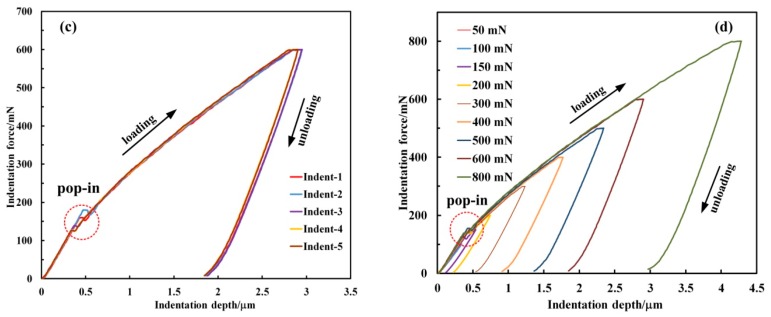
Indentation force-depth (*F*–*h*) curves. (**a**,**c**) are the five repeated *F*–*h* curves with peak force of 600 mN under the Berkovich and flat-tip indentation, respectively. (**b**,**d**) are the averaged *F*–*h* curves at different peak forces with the Berkovich and flat-tip indenters, respectively. Each single curve in (**b**,**d**) was obtained from the averaged data of the five repeated *F*–*h* curves at corresponding force.

Figure 3 shows the indentation force-depth (*F*–*h*) curves measured experimentally, where the parameter *F* designates the indentation force and *h* the indentation depth relative to the initial undeformed surface. As seen from Figure 3a,c, the five repeated *F*–*h* curves agree very well with each other. In Figure 3b,d, the loading curves under Berkovich indentation did not coincide with each other as well as those under flat-tip indentation because of the different loading rate in the experiment. On the other hand, the loading curves trend with a positive second derivative under Berkovich indentation while trending negatively under flat-tip indentation. This is because that the contact area increased as Berkovich indenter penetrating into the sample while it remained constant under flat-tip indentation [16]. In addition, an obvious pop-in phenomenon was always observed in the flat-tip *F*–*h* curves at a force of about 150 mN, as shown in Figure 3. The pop-in phenomenon characterized the transition from elastic to plastic material deformation during indentation loading. This elastic–plastic transition was also proven by the *F*–*h* curves and indent imprint observations at 50 and 100 mN of flat-tip indenter. In the 50 and 100 mN *F*–*h* curves, the loading and unloading curves were coincident and linear (see Figure 3d). On the other hand, the indent imprints at 50 and 100 mN were invisible under SEM which will be discussed later. In other words, only elastic deformations occurred at 50 and 100 mN. In contrast, no pop-in phenomenon was observed in the Berkovich *F*–*h* curves as reported in [10].

From the experimentally measured *F*–*h* curves, Young’s modulus and hardness of GaAs were calculated. For the flat-tip indentations at 50 and 100 mN, elastic contact Hertz model [17,18] was applied to estimate Young’s modulus as

(1) $$E_{\mathrm{eff}}=\frac{F}{2hr}$$

where *E*_eff_ is the effective elastic modulus defined by

(2) $$E_{\mathrm{eff}}={\left(\frac{1-\nu^{2}}{E}+\frac{1-\nu_{i}^{2}}{E_{i}}\right)}^{-1}$$

and *r* is the radius of flat-tip indenter. The effective elastic modulus takes into account the fact that elastic displacements occur in both the tested sample, with Young’s modulus *E* and Poisson’s ratio *v*, and the indenter, with elastic constant *E*_i_ and *v*_i_ (for the diamond indenters used in this report *E*_i_ = 1140 GPa, *v*_i_ = 0.07, and *v* = 0.3 for GaAs [10]).

Otherwise, Oliver and Pharr method [16,19] was used for the calculation of Young’s modulus and hardness. In this method, the unloading curves are approximated by the power law relation

(3) $$F=\alpha {\left(h-h_{r}\right)}^{m}$$

where *α* and *m* are power law fitting constants, *h*_r_ is the residual permanent depth of penetration after the indenter is fully unloaded. As mentioned previously, the contact area remained constant in the flat-tip indentation as the indenter was withdrawn. It resulted that the unloading curves in flat-tip indentation were linear as shown in Figure 3c,d. Therefore, in Equation (3) it is *m* = 1 for the flat-tip indenter.

The elastic unloading stiffness (also called the contact stiffness), *S* = d*F*/d*h*, defined as the slope of the upper portion of the unloading curve during the initial stages of unloading is expressed as

(4) $$S=m\alpha {\left(h_{\max}-h_{r}\right)}^{m-1}$$

where *h*_max_ is the maximum indentation depth in the *F*–*h* curves. Then the contact depth *h*_c_ along which contact is made between the indenter and the specimen is calculated by

(5) $$h_{c}=h_{\max}-\varepsilon \frac{F_{\max}}{S}$$

Hereby, *ε* is a constant depending on the indenter geometry (*ε* = 0.75 for Berkovich indenter, *ε* = 1.00 for flat-tip indenter) and *F*_max_ is the maximum indentation force in the *F*–*h* curves. In case of the Berkovich indenter, the contact area *A*_c_ is calculated as

(6) $$A_{c}=24.5h_{c}^{2}$$

and for the flat-tip indenter

(7) $$A_{c}=\pi r^{2}$$

Once the contact area is determined, measurement of the effective elastic modulus follows

(8) $$E_{\mathrm{eff}}=\frac{\sqrt{\pi }}{2}\frac{\beta S}{\sqrt{A_{c}}}$$

where *β* is a constant relating to indenter geometry (*β* = 1.034 for Berkovich indenter, *β* = 1.00 for flat-tip indenter). Then the Young’s modulus *E* can be induced by Equation (2).

For the hardness measurement, the hardness *H* is estimated from

(9) $$H=\frac{F_{\max}}{A_{c}}$$

**Figure 4:**
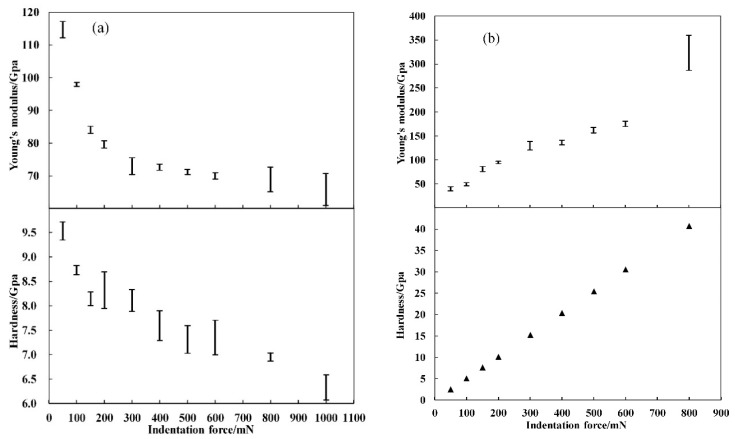
Measured Young’s modulus and hardness as functions of indentation force for (**a**) Berkovich indenter and (**b**) flat-tip indenter. The error bar means that the data was obtained from the five repeated measurement under one single force.

Based on the above calculation, the dependences of Young’s modulus and hardness on the indentation force are shown in Figure 4a,b for Berkovich and flat-tip indenters, respectively. As seen from Figure 4a, Young’s modulus decreased rapidly from 115 to 75 GPa with force from 50 to 300 mN and kept in the range of about 60–70 GPa with force from 300 to 1000 mN. In the case of hardness, it also decreased as the applied force were increased under Berkovich indentation with a range of 6–10 GPa. Under the flat-tip indentation, as shown in Figure 4b, Young’s modulus and hardness increased linearly with the applied force due to the constant indentation-induced contact area.

**Figure 5:**
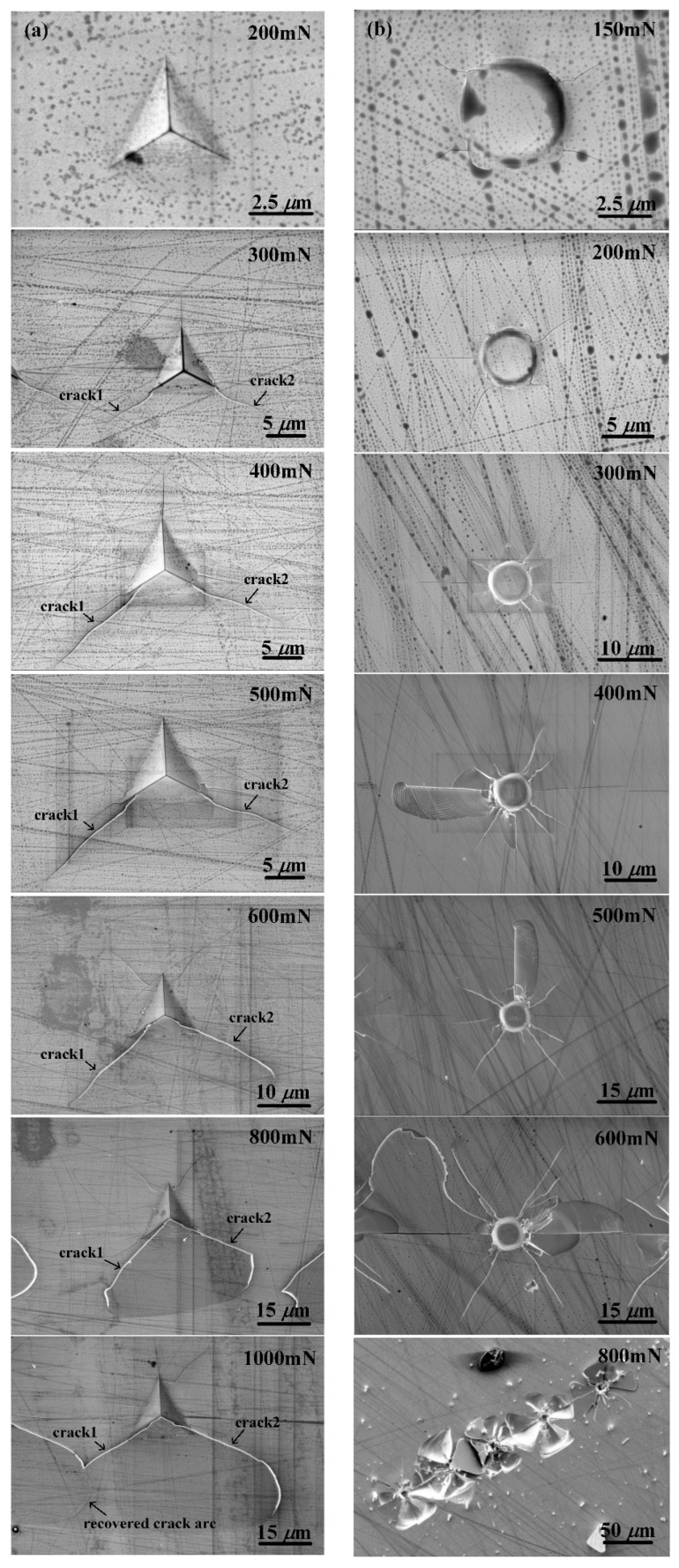
SEM images of typical indent imprints at different forces for (**a**) Berkovich indenter and (**b**) flat-tip indenter. “crack 1” and “crack 2” in (**a**) indicate two main cracks caused at different forces. “800mN” image in (**b**) gives the five crushed imprints at 800 mN under the flat-tip indentation.

Figure 5 gives the SEM images of typical indent imprints at different forces for Berkovich and flat-tip indenters. Under the Berkovich indentation, perfect pyramid indent imprints were made at the force of 50, 100, 150, and 200 mN. Two main cracks marked as “crack1” and “crack2” in Figure 5a arose at the edge end of the imprint from the force of 300 mN and the cracks length increased with force increasing. From the force of 800 mN, these two cracks developed with arcs at the end and tended to peel off the surface. A very interesting phenomenon was observed at 1000 mN force that the peeling crack arc of “crack2” of one imprint was recovered by “crack1” of the nearby imprint made by the next indentation, as illustrated in “1000mN” image in Figure 5a. It may probably suggest that the crack might be prevented and recovered by the nearby crack in the Berkovich indentation. 

**Figure 6:**
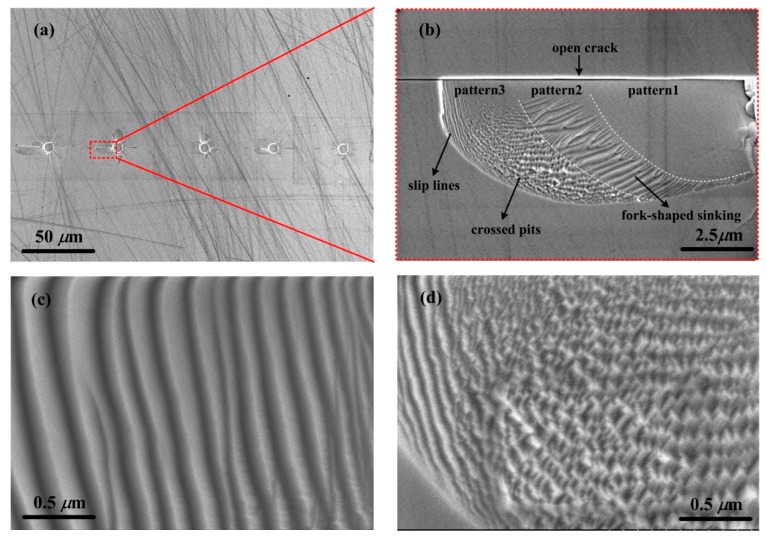
(**a**) SEM image of the five imprints after flat-tip indention of 400 mN. (**b**) Enlarged image of the wing-shaped sinking region along the open crack. The dashed curves indicate the boundary of three distinct surface material sinking patterns, named as “pattern1”, “pattern2”, and “pattern3”. (**c**,**d**) are the enlarged images of the slip lines and crossed pits marked in (**b**).

In the case of flat-tip indentation, no indent imprint was found at the force of 50 and 100 mN due to the elastic deformation as mentioned previously. Once plastic deformation took place from 150 mN, cracks appeared and spread around the indenter boundary edge. The lengths of cracks increased as the indentation force increased. From 400 mN, surface material sank into a shape of wing along some of open cracks as shown in Figure 5b. This wing-shaped sinking of surface material may probably be induced by the sharp shear strain generated under the boundary edge of flat-tip indenter by indentation [20,21]. Details of the wing-shaped sinking are shown in Figure 6. Three distinct patterns of material sinking appeared as separated as “pattern1”, “pattern2”, and “pattern3” by dashed curves in Figure 6b. In “pattern1” region, the surface material only sank with the same structural feature as the undeformed material. In “pattern2” region, the surface material sank in parallel fork shapes along the dashed boundary and the fork-shaped sinking diverged in both density and shape close to the open crack. In “pattern3” region, a large density of parallel slip lines and crossed pits were generated as shown in Figure 6b. The parallel slip lines were almost uniformly distributed as well as the crossed pits as shown in Figure 6c,d, respectively. The arising of the fork-shaped sinking, slip lines, and crossed pits on the sample surface may probably be due to the slip bands and converging crossed twinning deformations inside the material as reported in [12]. At the force of 800 mN, GaAs material was totally crushed in a symmetrical flower pattern as shown in “800mN” image in Figure 5b, relating to the rosette arm pattern of deformation of GaAs material [12].

Based on the above analysis, we can see that cracks appeared at force between 100 and 150 mN for flat-tip indentation while between 200 and 300 mN for Berkovich. Moreover, both material sinking and fatal destruction were observed under the flat-tip indentation. In other words, cracks and destructions on GaAs material surface took place more easily under the flat-tip indentation than that of Berkovich. Therefore, a machining tool with sharp tip is recommended for the mechanical machining of brittle materials like GaAs.

## 4. Conclusions

In conclusion, five main findings emerged from this work. Firstly, the loading curves trend with a positive second derivative under Berkovich indentation while negative under flat-tip indentation because of the contact area changes during loading. An obvious pop-in phenomenon representing elastic–plastic transition always occurred at the force of about 150 mN with the flat-tip indenter while it did not show up under Berkovich indenter. Secondly, the Young’s modulus and hardness decreased as the applied force were increased under Berkovich indenter with ranges of 60–115 GPa and 6–10 GPa, respectively, while they increased linearly with the applied force under flat-tip indentation due to the constant contact area. Thirdly, obvious cracks took place from the force of 300 mN at the edge end of indented imprint by Berkovich indenter and the crack length increased with the applied force. Cracks developed with arcs at the end from 800 mN and be recovered by the nearby imprint made by the next indentation from 1000 mN. This may probably suggest that the crack might be prevented and recovered by the nearby crack in the Berkovich indentation. Fourthly, in the case of flat-tip indentation, cracks appeared and spread around the indenter boundary edge once plastic deformation took place from 150 mN. From 400 mN, surface material sank into a shape of wing with three distinct patterns along open cracks. In the wing-shaped sinking region, a density of fork-shaped sinking, slip lines, and crossed pits were generated and they may probably be contributed to the slip bands and converging crossed twinning deformations inside the material. Lastly, a machining tool with sharp tip is recommended for the mechanical machining of brittle materials like GaAs because cracks and destruction on GaAs material surfaces took place more easily under the flat-tip indentation than that of Berkovich.
